# Experience of a novel lung transplant program in Mexico

**DOI:** 10.3389/frtra.2024.1347603

**Published:** 2024-07-23

**Authors:** Mariana N. Zavala-Gómez, Patricia Rodríguez-de la Garza, Uriel Chavarria-Martinez, Manuel Wong-Jaen, Adrián Camacho-Ortiz, Lilia M. Rizo-Topete, Alicia E. López-Romo, Vicente Fuentes-Puga, Sergio S. Sánchez-Salazar

**Affiliations:** ^1^ Hospital Christus Muguerza Alta Especialidad, Grupo Christus Muguerza, Monterrey, Mexico; ^2^ Ciencias de la Salud, Universidad de Monterrey, Monterrey, Mexico; ^3^ Lung Transplant Program, Christus Muguerza Alta Especialdiad, Monterrey, Mexico; ^4^ Infectology Lung Transplant Program, Christus Muguerza Alta Especialdiad, Monterrey, Mexico; ^5^ Nephrology Lung Transplant Program, Christus Muguerza Alta Especialdiad, Monterrey, Mexico; ^6^ Pulmonary and Critical Care Medicine, Lung Transplant Program, Christus Muguerza Alta Especialdiad, Monterrey, Mexico

**Keywords:** lung, transplant, experience, Mexico, allocation

## Abstract

Lung transplantation is the gold standard therapy for patients in the end stages of pulmonary disease. However, in numerous countries, candidates for lung transplants often die on the waiting list due to a shortage of donors and limited access to transplant centers. This article delves into the experience of our hospital, Christus Muguerza in Monterrey, Mexico, as the sole active lung transplant program in the country, having conducted 35 transplants from August 2017 to March 2023. We discuss the actual situation of lung transplantation in Mexico and the challenges we have faced over time, such as late patient referrals for evaluation and eventual transplantation. In addition, we outline the challenges we anticipate as more transplant programs emerge in the country.

## Introduction

Lung transplantation is a viable treatment option for patients suffering from chronic end-stage lung diseases. Available since the 1980s, nearly 4,000 lung transplants are performed worldwide each year (1). According to the latest report from the International Society for Heart and Lung Transplantation (ISHLT) registry, the number of lung transplants has increased steadily over time. In the 1990s, there were nearly 11,000 transplants, around 22,000 in the 2000s, and almost 34,000 between 2010 and 2018 (2, 3). This increase is due to continuous improvements in donor selection, recipient processes, surgical and anesthetic techniques, immunosuppression, and post-transplant care.

Lung transplant is now an established treatment option for patients with various end-stage lung diseases (1). However, in many countries, lung transplant candidates die on the waiting list due to a shortage of donors and limited access to transplant centers. Therefore, the importance of an efficient donor organ allocation system to reduce mortality and improve transplant outcomes. Currently, the rules guiding allocation in most countries are based on urgency and transplant benefit, with survival benefit being the primary goal (4).

However, evaluating the effects of allocation systems on lung transplantation, identifying systemic flaws, and revising the systems, especially in low-transplant-volume countries, are challenging (4). This issue is significant in Mexico, which has limited experience in lung transplantation with slow development.

The first lung transplant in Mexico dates back to 1989; nonetheless, before 2003, there was no structured program in the country. According to some articles, but not confirmed by any official transplant organization, the last lung transplants registered in Mexico were performed in 2006 by the Instituto Nacional de Enfermedades Respiratorias and the Centro Medico Nacional Siglo XXI, without success (5). After this date, there is only reference to 12 transplants performed countrywide, all of them in the city of Monterrey (6).

## Birth of a program

In 2017, the Christus Muguerza lung transplant program was founded in Monterrey, and it currently stands as the only active lung transplant program in the country. To date, this program has performed 35 transplants.

This program is relatively new, boasting only 6 years of experience and a rather limited population of lung transplant recipients. This limited sample size poses a challenge when attempting to draw meaningful comparisons with high-volume transplant centers. As a country with a nascent history in lung transplantation and the adoption of relatively recent protocols, our primary objective is to glean insights and knowledge from the more established and larger transplant centers.

Nevertheless, a persistent issue it faces is the inadequate supply of donor organs, which falls far short of meeting the needs of patients who stand to benefit from this life-saving procedure. Mexico has a deceased donor rate of only 4.3 per million inhabitants, significantly lower than the average of 8.2 per million inhabitants for Latin America (7). According to the latest data from 2023, the National Center of Transplant of Mexico (CENATRA) reported that 19,962 patients are on the waiting list, with renal transplants (16,370) leading the list, followed by the liver (204), heart (24), and lung (2). They reported that the most common solid organ transplant was renal, with 3,082 transplants, 918 of which were from deceased donors. The challenge is even greater in lung transplantation, as there is no national registry for the number of lung transplant donors maintained by the National Transplant Center of Mexico (8).

In Mexico, there is currently no established protocol for lung transplant selection. For this reason, we have adopted protocols from international transplant centers, which report using the lung allocation score (LAS) as an allocation method. This score intends to maximize the benefit for recipients by reducing waiting list mortality (9, 10). It predicts the urgency (1-year survival without a transplant) and survival (1-year survival with a transplant) measures. Priority is given to patients with the highest scores within local and regional organ procurement organizations based on the LAS score.

Previous research has shown that patients with LAS scores greater than 46 are at an increased risk of death (11). However, different cutoff points have been used based on the center's experience and studies evaluating the association between LAS and post-transplant survival.

## Our experience

Describing our brief experience, our cohort included 35 transplant patients from August 2017 to March 2023. The median age of transplant recipients was 60 (range 46–67) years. Among our patients, 24 (68.5%) were men. Double lung transplantation was performed in 26 patients (74.2%). Single lung transplantation was performed in nine due to the high risk of mortality and complication**s**. The causes of lung disease included idiopathic pulmonary fibrosis (60%) and *sequelae* of COVID-19 (14.2%). Other causes included patients with chronic obstructive pulmonary disease (COPD), pulmonary arterial hypertension, lymphangioleiomyomatosis (LAM), and cystic fibrosis. Seven recipients were bridged through extracorporeal membrane oxygenation (ECMO) before transplantation, with a median duration of 49 days.

Our standard immunosuppression regimen consists of basiliximab for induction and tacrolimus and mycophenolate mofetil for maintenance. Describing the transoperative and postoperative management, the median surgical time was 416 (351–495) min and the median ischemia time was 303 (225–360) min. Out of 35 patients, 8 (22.8%) required ECMO transoperative support, especially veno-pulmonary arterial support; after the procedure and 15 (42.8%) required ECMO support, mostly veno-venous, except for 1 patient with pulmonary hypertension who required venoarterial (VA) support. After lung transplantation, the duration of mechanical ventilation was 8 days [interval reference (IR): 8–32 days], with 9 days (IR: 9–31 days) spent in the ICU and a hospitalization duration of 24 days (IR: 22–57 days).

As part of the candidacy evaluation, the LAS is calculated at the beginning of the transplant protocol. We do not perform any updates or recalculations of the LAS score, as the average waiting time on our transplant list is relatively short, at 28 (15–45) days. With zero mortality on the waiting list, every patient admitted to the protocol undergoes transplantation. The absence of mortality could be explained by our small population, which consists of the fortunate few patients who have access to lung transplants through private healthcare institutions, either due to medical insurance or economic resources. As we mentioned, we are the only active lung transplantation center, so most of the donated organs are allocated to our program and come from both public and private hospitals. Only one public institution in our region has recently begun to adopt the protocol.

Patients from our cohort had a mean LAS score of 50.34 (SD 17.87). There was variability in the LAS score among the four etiologies described: patients with COPD had a mean score of 37.5, patients with cystic fibrosis had a mean score of 40.6; patients with restrictive diseases such as idiopathic pulmonary fibrosis (IPF) had a mean score of 48.29, and patients with pulmonary vascular disease had a mean score of 60 (Table 1). Another significant group of patients in our cohort consisted of COVID-19 patients. Patients with SARS-COV-2 pneumonia have emerged as a novel etiological group for lung transplantation; although this pathology is not included, there have been reports in the LAS score in some centers. Their inclusion in a transplant protocol with a high LAS score is explained by the extended hospitalization and typical complications associated with COVID-19. We highlight a score for COVID-19 patients of 70.9, which is similar to those reported in some centers, such as 74.7 (33.1–94) recorded by King et al. (12).

**Table 1:** Demographic and clinical characteristics.

Variable (*n* = 35)	
Male (%)	24 (68.57)
Age (DS)	60 (50.83)
Double lung transplant (%)	26 (74.28)
Etiology (%)	
IFP	21 (60)
COVID-19	5 (14.28)
COPD	2 (5.71)
PH	2 (5.71)
LAM	1 (2.85)
CF	1 (2.85)
Others	3 (8.57)
LAS (SD)	50.34 (17.87)
IFP	48.29 (13.68)
COVID-19	70.9 (28.95)
COPD	37.5 (4.94)
PH	63 (0)
LAM	32.8 (NA)
CF	40.6 (NA)
Others	39.64 (8.45)
ECMO (%)	
Preoperative support (days)	7 (20)
Transoperative support (days)	8 (22.8)
Postoperative support (days)	15 (42.8)
Transoperative characteristics (IR)	
Surgical time (min)	416 (351–495)
Ischemia time (min)	303 (225–360)
Postoperative care (IR)	
Mechanical ventilation (days)	8 (8–32)
ICU stay (days)	9 (9–31)
Hospitalization (days)	24 (22–57)

SD, Standard Deviation; IPD, idiopathic pulmonary disease; PH, pulmonary hypertension; CF, cystic fibrosis.

It is worth noting that the mean LAS score of our patients surpasses the threshold associated with an elevated risk of mortality, set at 46. The factor mostly associated with a higher LAS score in our cohort is the late reference of the patients to our lung transplant program by their medical teams. Patients with an LAS score of ≥46–59 encompassed 25% of our cohort, while those scoring 60–79 constituted 8% and ≥80 accounted for 12%. Most of our patients were referred with advanced stages of their disease, multiple comorbidities, and worse baseline lung function; therefore, they had high LAS scores.

Several major complications frequently arise following LT that commonly lead to significant morbidity and mortality. They are considered in the context of time periods; in the first year, they are related to events occurring during the transplant procedure and the postoperative phase, the first 30 days. The factors associated with operative and perioperative bleeding, anastomotic complications, ventilator-induced barotrauma, and the inevitable complication of infection may all contribute, either singly or in combination, to allograft dysfunction and failure and consequent death (3, 13).

The overall 30-day survival rate was 65.7%. One patient died during surgery. The main causes of death were infection (22.8%) and chronic rejection (11.4%).

Primary graft failure (PGF) is ascribed to poor function of the allograft, stemming from the combined processes of organ procurement, implantation, and reperfusion (13). It represents 10%–30% of early mortality and morbidity (13, 14). PGF was observed in nine (25.7%) of our lung transplant patients, causing early mortality in four of our lung transplant patients (36%) vs. five cases (20%, *p* = 0.007). This percentage of early mortality associated with PGF appears significant in our analysis.

## Future of a new challenge

During the time our program has been active, we have faced numerous challenges. One of them is the late referral of the patients for evaluation and eventual transplantation. Because of the past absence of lung transplant programs in Mexico, there was no culture of lung transplantation as an alternative. The few Mexican patients who could go abroad received transplants in the United States, while some were treated in Spain. Introducing the program required convincing the medical community that lung transplantation is a viable option in Mexico and that it implies a significant team effort with prompt timing for referral and long-term care.

Over the years, our team has actively engaged in numerous scientific discussions with the medical community to advocate for lung transplants. As a result, we are proud to report that at least three additional hospitals in Mexico are currently in the process of establishing their own dedicated programs. This expansion is particularly crucial in our large country, where the substantial population demands multiple lung transplant initiatives. Our ongoing efforts have successfully demonstrated the feasibility of lung transplants, solidifying their position as a viable and effective medical procedure within our nation.

The new programs will face the challenge of donor scarcity, making the LAS score relevant for appropriate allocation. Envisioning a collaborative effort, we hope that all programs, including ours, can work together to cultivate a fresh mindset within the medical community. This shared goal involves fostering a culture of appropriate and timely patient referrals, as well as enhancing the overall culture of organ donation. All of this requires a team effort and continuous medical information, including training abroad in the field.

## Conclusion

In conclusion, Mexico still faces significant cultural and logistical obstacles in the realm of transplantation, such as the lack of a well-established reference protocol, limited experience in lung transplantation, low availability of air transportation leading to longer cold ischemia times, socioeconomic disparities in access to transplantation services, lack of *ex vivo* lung perfusion programs, and the persistence of cultural factors and misconceptions about organ donation. Despite these challenges, it is noteworthy that Mexico has made improvements in developing its lung transplantation program, and the experiences gained from these procedures can contribute to improving the programs and patient outcomes. It is evident that as more programs are initiated, the adequate allocation of lungs to each program will become a challenge for our country.

## Data Availability

The raw data supporting the conclusions of this article will be made available by the authors without undue reservation.
